# TEA: the epigenome platform for Arabidopsis methylome study

**DOI:** 10.1186/s12864-016-3326-6

**Published:** 2016-12-22

**Authors:** Sheng-Yao Su, Shu-Hwa Chen, I-Hsuan Lu, Yih-Shien Chiang, Yu-Bin Wang, Pao-Yang Chen, Chung-Yen Lin

**Affiliations:** 1 Bioinformatics Program, Taiwan International Graduate Program, Institute of Information Science, Academia Sinica, Taipei, Taiwan; 2 Institute of Information Science, Academia Sinica, Taipei, Taiwan; 3 Institute of Biomedical Informatics, National Yang-Ming University, Taipei, Taiwan; 4 Institute of Plant and Microbial Biology, Academia Sinica, Taipei, Taiwan; 5 Division of Biostatistics and Bioinformatics, Institute of Population Health Sciences, National Health Research Institutes, Zhunan, Miaoli Taiwan; 6 Institute of Fisheries Science, College of Life Science, National Taiwan University, Taipei, Taiwan

## Abstract

**Background:**

Bisulfite sequencing (BS-seq) has become a standard technology to profile genome-wide DNA methylation at single-base resolution. It allows researchers to conduct genome-wise cytosine methylation analyses on issues about genomic imprinting, transcriptional regulation, cellular development and differentiation. One single data from a BS-Seq experiment is resolved into many features according to the sequence contexts, making methylome data analysis and data visualization a complex task.

**Results:**

We developed a streamlined platform, TEA, for analyzing and visualizing data from whole-genome BS-Seq (WGBS) experiments conducted in the model plant *Arabidopsis thaliana*. To capture the essence of the genome methylation level and to meet the efficiency for running online, we introduce a straightforward method for measuring genome methylation in each sequence context by gene. The method is scripted in Java to process BS-Seq mapping results. Through a simple data uploading process, the TEA server deploys a web-based platform for deep analysis by linking data to an updated *Arabidopsis* annotation database and toolkits.

**Conclusions:**

TEA is an intuitive and efficient online platform for analyzing the *Arabidopsis* genomic DNA methylation landscape. It provides several ways to help users exploit WGBS data.

TEA is freely accessible for academic users at: http://tea.iis.sinica.edu.tw.

## Background

Genomic DNA methylation is a long observed phenomenon. It was first described in prokaryotes and was found as a defense system of the genome against foreign DNA invasion (e.g., R-M system, [1]). In eukaryotes, it is known to play roles in regulating gene activity [2–4]. DNA methylation can be a stable change, such as genomic imprinting in diploid organisms to label the parental origin of the genome constituents in the zygote or to regulate gene dosage in sexual dimorphic chromosomes. Recent studies reveal that genomic DNA methylation can be changed dynamically; working together with histone codes, DNA methylation is recognized as an epigenetic marker, i.e., to alter genome activity without changing the sequence context [5–7].

5-methylcytosine (5 mC) is the best-characterized methylation type in genomic DNA. It can be classified into three different sequence contexts by the neighboring bases, CG, CHG, and CHH (H = A, C, or T). In plants, CG methylation is maintained by DNA methyltransferase 1 (MET1), the plant homolog of DNMT1 [8–12]. The plant-specific DNA methyltransferase chromomethylase 3 (CMT3) activity promotes CHG methylation, which is linked to the histone H3 lysine 9 (H3K9) dimethylation condition [13–15]. Asymmetric CHH methylation is mediated by two DNA methyltransferases: chromomethylase 2 (CMT2), and domains rearranged methyltransferase 2 (DRM2) [16]. CMT2 mainly functions at the pericentromeric and long transposable element region, and DRM2 mediates CHH methylation through the RNA-directed DNA methylation (RdDM) pathway [17, 18]. The fact that methylation of cytosine in different sequence contexts is maintained/modified by different enzymes and pathways implies complex regulation of genomic methylation status and attracts researchers interested in various subjects.

Bisulfite sequencing (BS-Seq) has become a standard technology to profile genome- wide DNA methylation at single-base resolution. Briefly, genomic DNA is treated with sodium bisulfite before high-throughput next generation sequencing (NGS). Bisulfite modification converts non-methylated cytosines to uracils (read as T in the sequencing reaction) while methylated cytosines remain unchanged. The frequency of C/C + T at each C is calculated from reads mapped to the position to give the cytosine methylation measurement at each C in the genome. Tools have been developed to deal with whole-genome BS-Seq (WGBS) data, such as BiSeq [19], BSmooth [20], DMAP [21], methPipe [22], methylKit [23], methylPipe [24], methylSig [25], MOABS [26], radMeth [27], and WBSA [28]. Most of these analysis tools focus on identifying differentially methylated regions (DMRs) and defining differentially methylated genes (DMGs) as genes with DMRs in or near the gene body.

*Arabidopsis thaliana* is a popular model plant. It is a selfing (or self-fertilization) species with a small and completely sequenced genome from the standard strain Col-0. The genome of *A. thaliana* is comprised of five nuclear chromosomes plus mitochondrial and chloroplast DNA. The present genome build, TAIR10, is 135 Mb. Arabidopsis has been used in a wide range of studies which are collected in a centralized resource [29]. One special interest in studying plant genomes is the relationship between DNA methylation and transposable element silencing. In plants, most DNA methylation occurs at transposable elements and other repetitive DNA sequences. The DNA methylation pathway is described as a powerful tool for flowering plants to silence these high amounts of transposon parasites in their genomes [30]. Recently, a comprehensive survey on the Arabidopsis methylome published a whole series of BS-Seq data from a panel of gene silencing mutants at single-nucleotide resolution [31]. Reanalyzing the openly accessible methylome data can serve as a good starting point for bench scientists to conceive of new studies; however, it is often hampered by a ceiling on data-handling skills. Various features according to the sequence contexts and locations need to be resolved and compared. The workflow for a BS-Seq data analysis process is more complicated than a transcriptome workflow.

In this paper, we present an intuitive data analysis platform for Arabidopsis whole-genome bisulfite sequencing (WGBS) data, TEA (**T**he **E**pigenomic platform for **A**rabidopsis, http://tea.iis.sinica.edu.tw). To crop the essence of genome methylation status and to meet the efficiency for performing analysis online, we introduce a straightforward method for measuring genome methylation level in each sequence context by gene. This method is implemented in an in-house program (*EpiMolas.jar*) to process BS-Seq mapping results from **CGmap** by BS-Seeker2 [32] and **CX_report** by Bismark [33] into a small, tab-delimited data file, *mtable*. The *mtable* upload process triggers a website deployment by linking data files to gene annotation databases (TAIR10, KEGG, GO etc.) and to versatile data analysis and display modules in TEA. Using this user-friendly interface, the summary of gene and promoter methylation levels among experiment conditions are retrieved and analyzed easily.

## Methods

### The measurement of methylation level

The methylation landscape in TEA is based on the gene, in which DNA methylation levels in the promoter and gene body are estimated from the WGBS data. The DNA methylation level for individual cytosines is estimated as Equation (1.1):

1.1 $$ \mathrm{the}\ \mathrm{D}\mathrm{N}\mathrm{A}\ \mathrm{methylation}\ \mathrm{level}\ \mathrm{f}\mathrm{o}\mathrm{r}\ \mathrm{individual}\ \mathrm{cytosine}\ i= Ci = \frac{\#\  read\ C}{\#\  read C+ readT} $$

Calculating the average promoter or gene body methylation levels within the promoter or gene body is then the average *C*_*i*_ within the range by Equation (1.2):

1.2 $$ \mathrm{Average}\ \mathrm{D}\mathrm{N}\mathrm{A}\ \mathrm{methylation}\ \mathrm{level}\ \mathrm{in}\ \mathrm{promoter}\ \mathrm{or}\ \mathrm{gene}\ \mathrm{body} = \frac{{\displaystyle \sum_{i\in X}{c}_i}}{{\displaystyle \sum_{i\in X}1}} $$

X = promoter or gene body

We count the number of reads mapped to each C with a minimum threshold of four, making an average methylation percentage of at least five C sites of each sequence context type, and give six measurements (i.e., pmt-CG, pmt-CHG, pmt-CHH, gene_CG, gene_CHG, gene_CHH) of a gene. Whole Genome Bisulfite sequencing is actually considered the gold standard approach for profiling genome-wide DNA methylation [34], and has become the standard profiling method in major epigenome consortiums, such as NIH Roadmap [35], ENCODE [36], Blueprint [37], and IHEC [38]. The major sources of error originate from DNA sequencing errors and incomplete bisulfite conversion. The former is now well controlled, with the advancement of NGS, and is generally <0.01%. The latter is usually >99.5% in a stable lab practice. When a region such as a promoter or gene body is analyzed, TEA requires at least five cytosines to be included in order that any potential bias from individual cytosines can be minimized.

**Fig. 1:**
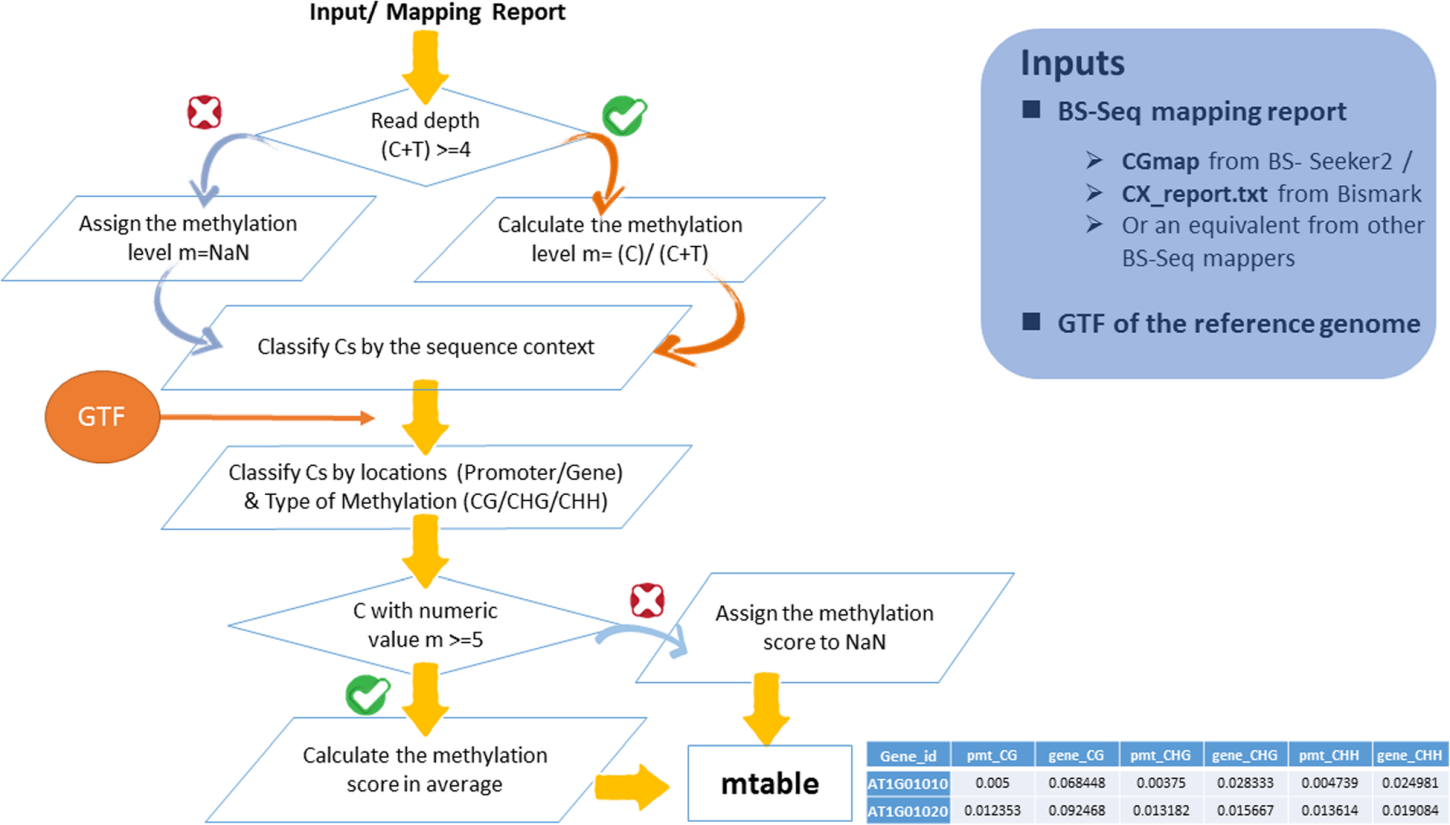
Flowchart of methylation level extraction in EpiMolas.jar. EpiMolas.jar accepts output from several types of BS-seq mapping reports and generates six methylation profiles in three contexts, CG/CHG/CHH, and two locations, promoter/gene body, based on a gene annotated file

The methylation level is thus a normalized score from 0 (all observed sites are unmethylated) to 1 (all observed sites are methylated), or “NaN” for genes that do not have sufficient reads/sites to calculate the index. The overall calculation process is shown in Fig. 1.

The method is scripted in Java to process BS-Seq mapping results, e.g., *.CGmap from BS SEEKER2 [32] or CXreport.txt from Bismark [33]. This program *EpiMolas.jar* (download from link in http://tea.iis.sinica.edu.tw/tea/mtable.html) also requires the *Arabidopsis* gene annotation file (gtf) to calculate the methylation measurement file ***mtable. mtable*** is a tab-delimited pure text file, indexed by gene identifiers and six measurements of methylation levels. It is the upload format for the TEA server. Processes for executing *EpiMolas.jar* to generate ***mtable*** are described in TEA online help.

### System implementation

TEA (**T**he **E**pigenomic platform for **A**rabidopsis) is constructed using LAPP system architecture (Ubuntu 14.04, Apache 2.04, PostgreSQL 9.1, and PHP 5.1) with bootstrap 3 CSS framework (http://getbootstrap.com/) to provide an intuitive user experience. The whole system runs in a virtual machine (CPUs of 2.27 GHz, four cores, 16 GB RAM and 500 GB storage) on the cloud infrastructure of the Institute of Information Science, Academia Sinica, Taiwan. The TEA annotation database includes genome structure and gene annotation for *A. thaliana*. It based on Ensembl (Ensembl Plants release 32) and function-rich annotations like GO terms from Gene Ontology (Aug. 2016) and KEGG Pathways (Apr. 2016). Data retrieving, integration and real-time calculation in the analysis process are implemented in scripts written in Java (OpenJDK 7), Python (version 2.7), and PHP (version 5.1). Graphical visualization used HTML Canvas and SVG library to provide a high level of data interactivity. Biodalliance genome browser (version 0.13.7) [39] and Circos software package (version 0.69) [40] were integrated for browsing the genome structure and for accessing a gene list of chromosome coordinates, respectively.

### Heat map for 2D presentation in color

The heat map plot is implemented in java with jquery (version 2.1.4) and d3.js libraries (version 3.5.17, https://d3js.org/). Tree topologies of the dataset grouping and the methylation level of the subjects (promoter or gene body for a selected C context) are calculated by the SINGLE_LINKAGE in EUCLIDIAN_ DISTANCE method. We added tooltip functions to enhance the legibility as well as to access source data intuitively. For example, we introduced CanvasRenderingContext2D.drawImage with d3 to zoom and pan the heat map on the gene name (the Y axis on a heat map) or on the dataset label (the X axis on a heat map). Users can select a subset of genes from the heat map by mouse clicking on the gene name and saving the list for the module “Gene List Analysis.”

### Venn diagram for all possible logical relationships

For showing and counting all possible logical relationships between a finite collection of different sets, we implemented a Venn plot generator to render a diagram. It is a pure java script developed by our team to generate SVG without dependency on jquery or other libraries. This plot is also an interactive visualization tool to assist in subset selection without sophisticated Boolean operation. The implemented Venn plot function can generate comparison results for up to four selected sets. All of the possible relationships among different gene sets can be saved as a new gene list to module “Gene List Analysis” for deciphering deeper biological meanings.

### Gene Ontology terms and KEGG pathway enrichment calculation

Functional enrichment analysis pipelines are built in TEA for detecting overrepresented GO terms and KEGG entities in the given gene list compared with a random sampling from a whole-genome background. Customized P-value is an applicable cutoff for displaying enriched GO terms or KEGG pathways. The gene set enrichment score is measured using the hypergeometric test in Equation (2):

2 $$ \mathrm{p}\ \left(x\Big|N,m,n\right) = \frac{\left(\begin{array}{c}\hfill m\hfill \\ {}\hfill x\hfill \end{array}\right)\left(\begin{array}{c}\hfill N-m\hfill \\ {}\hfill n-x\hfill \end{array}\right)}{\left(\begin{array}{c}\hfill N\hfill \\ {}\hfill n\hfill \end{array}\right)} $$

where *x* is the number of genes in the test set in a particular annotation class (e.g., a given GO term), m is the gene number of the test set, N is the number of total genes being annotated in the whole sample space (i.e., the genome), n is the number of the genome in the given annotation class.

#### Demo datasets

The demo datasets are from Zhong et al. [41], which includes five previously published WGBS datasets, GSM881756, GSM1193638, GSM981015, GSM981017, and GSM981040 [31, 42]. All of these bisulfite sequencing reactions were carried out in 50-mer single-end format using an Illumina HiSeq2000. Raw read data files were downloaded from SRA/NCBI and were preprocessed with Cutadapt [43] to remove TruSeq adaptors. Cleaned reads were mapped to TAIR10 genome using BS Seeker2 [32] and Bismark [33]. The output files (*.CGmap /BS Seeker2 and CX_report.txt) were then converted to methylation data files (*.mtable) using ***EpiMolas.jar*** as described previously.

## Results

### Deploying the working platform

**Fig. 2:**
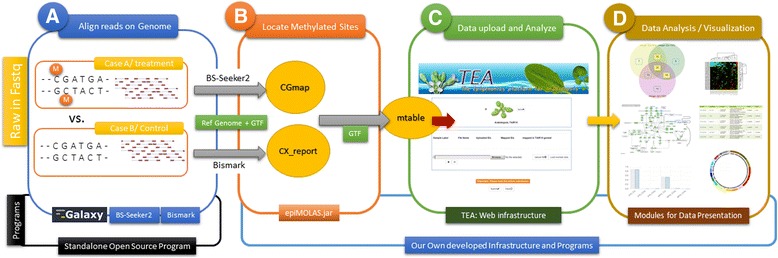
The workflow of TEA from raw reads to data visualization and deep analysis. **a** Align BS-seq read to the reference genome. **b** Generate mtable from CGmap, the output of BS Seeker2, or CX_report, the output of Bismark. **c** The main portal of TEA for data submission. **d** Data visualization with Venn diagram, boxplot, histogram, heatmap, and Circos plot

A working platform in TEA starts with a data uploading process. Users should make their mtable first (described previously). A step-by-step process guides users to define and check the overall data-to-TEA database mapping conditions in real time. The data deployment will take a few minutes to complete. Measurements on promoter regions of RNA genes (rRNAs, pre-tRNA, snRNAs, snoRNAs, miRNAs, and other RNA genes) and transposable genes (TE) are not included on the TEA-derived website. The whole process of TEA is illustrated in Fig. 2. Data-uploading details can be viewed through the demo tutorial on “New Submission”. Unregistered users can upload the data to TEA and run “have a trial” to get the analysis in a dynamic URL for 1-month access.

A summary of data mapping condition and usable measurements for each dataset and each methylation index are logged in the data website “home”. User should check this summary first. Unexpected values, such as a low gene id mapping rate or a low percentage of analyzable genes/promoters, are warning signs of problems in the data, for example, improper files were used in the data preprocessing steps or a low read throughput were used for resolving the methylome.

The basic data display view in TEA is the gene-centric page containing gene information, the methylation profile on CG, CHG, and CHH contexts of the gene (gene body) and the promoter region (−2500 to +500), an embedded genome browser, and gene function annotation (GO terms and KEGG pathways). The neighboring gene function allows users to browse the genes located up to a 5-Mb flanking range.

### Data modules

**Fig. 3:**
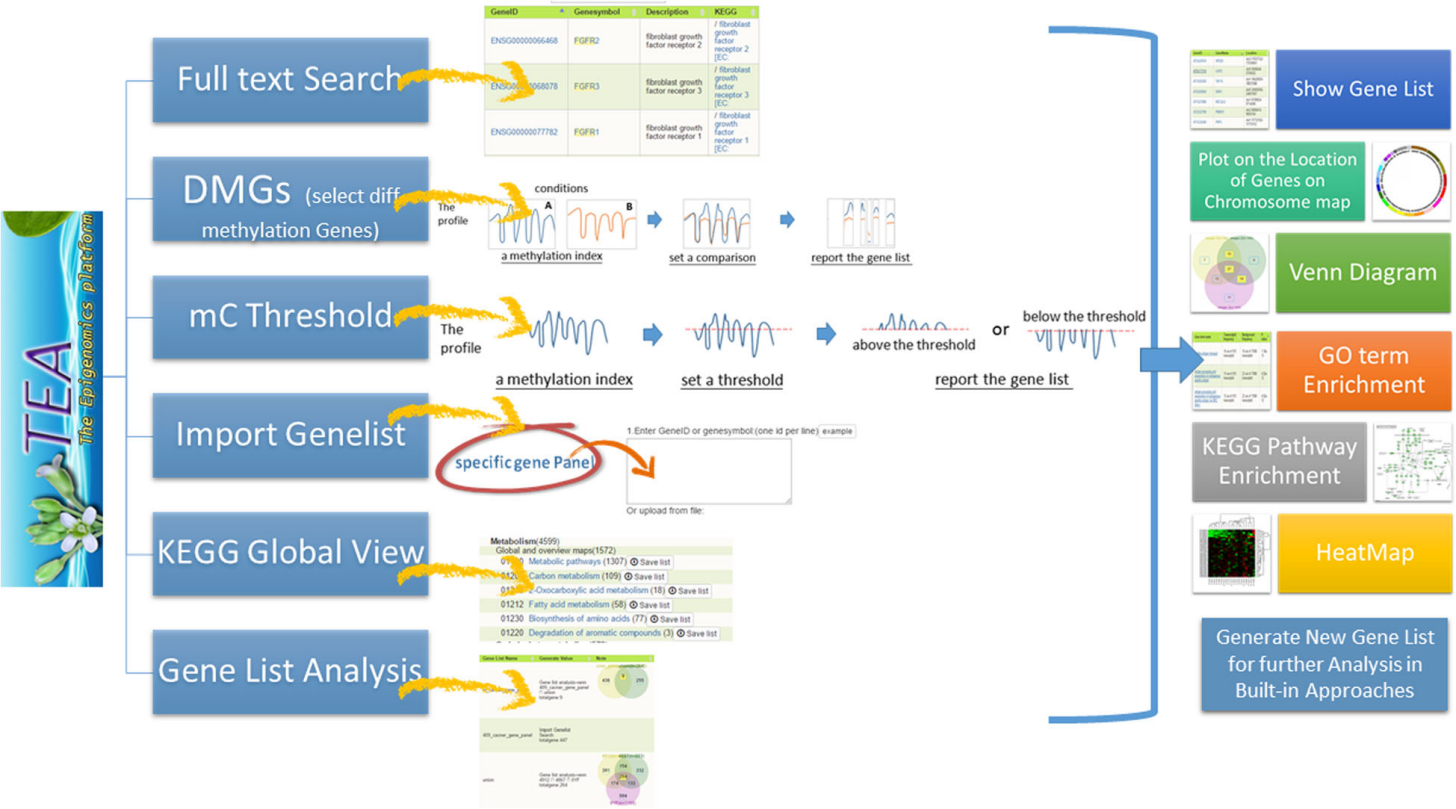
Six data modules in TEA. The full text search module is to search for genes of interest in the keyword search from the context of gene ID, gene symbol, gene description, and KEGG description. The DMGs module is to select differentially methylated genes (DMGs) on the basis of customized criteria. The mc Threshold module is to select DMGs by a cutoff value. The Import Genelist module is to upload lists of genes of interest. The KEGG Global View module is to display which genes are involved in each category of KEGG pathways. The gene list analysis module is to view the gene set from different analytic approaches including Venn diagram, heatmap, Circos plot, GO terms, and KEGG pathway enrichment analysis

TEA provides several ways to help users exploit their uploaded high-throughput data (Fig. 3). The data modules include the word search function on the annotation table, the gene ID list search function, the quantitative analysis functions on the differences (subtraction) or a threshold (cutoff) of a methylation measurement, and the canonical view in pathways. The gene list from all of these data retrieval steps can be kept as a gene list for later uses.

- Find Genes by Text
  In the module **Full Text Search**, users can find genes by Gene ID, gene symbol, gene description, and KEGG pathway description from the integrated annotation table in TEA. Further constraints on the gene biotypes and chromosome location can be applied to refine the search simultaneously. In the module **Import Genelist** query, users can paste or upload a file of a list in TAIR10/ ensembl gene IDs or gene symbols and obtain the matches. In the module **KEGG GlobalView**, users can browse genes on pathway maps. Items in a given map can be saved as a gene list for further analysis.
- Find Genes by Values
  Module **DMGs** (differentially methylated genes) is a pairwise comparison workflow for two data pools (experimental conditions) to which a single or multiple datasets can be assigned. Through a customized and flexible parameter setting, genes that fulfill the criteria are selected and can be further constrained on particular gene biotypes and chromosome locations. Module **mC Threshold** is used for selecting genes above or below a cutoff in at least one or among all of the selected datasets. These two modules provide different ways to access methylation landscapes in six different sequence contexts.
- Gene List Analysis
  Gene list items are derived from text search, id search, or a value comparison process and are kept for users’ needs. With the aid of the data displaying approach “show Venn diagram”, overlapping of elements (i.e., genes) in different gene lists is easily solved. Further manipulation on Boolean algebra, such as union, intersection, or other combinations, can be carried out on the interactive diagram to generate a new gene list.

### Data displaying approaches

We designed several ways to deepen the view of a gene list including “Show gene list”, “Plot on the location of genes on chromosome map” “Calculate GO term enrichment”, “Calculate KEGG pathway enrichment”, and “Draw heatmap with 2D clustering”. As mentioned in previous paragraphs, genes can be selected for different reasons via different data modules. Using “show gene lists”, the selected genes can be saved as a GeneList item for later uses. The approach “Show Venn Diagram” is only available in module **GeneList Analysis** to help on sub-list selection.

### Demo case: BS-Seq data reanalysis

To help users become familiar with TEA, we provide a demo dataset from Zhong et al. (2015) entitled “DOMAINS REARRANGED METHYLTRANSFERASE3 controls DNA methylation and regulates RNA polymerase V transcript abundance in Arabidopsis” [41]. Briefly, this study focused on elucidating the function of DRM3 (domain rearranged methyltransferase 3) in the RdDM pathway, which contains a catalytically inactive enzyme domain, but is required for *de novo* DNA methylation in vivo. The major conclusions from this study are: 1. DRM3 has moderate effects on global DNA methylation and 2. DRM3 interacts with Pol V, implying regulatory roles of Pol V- involved RNA-directed DNA methylation.

Five BS-Seq libraries were prepared from 3-week old leaf for detecting genome-wide methylation status under four conditions: two biological replicates in the control group and one dataset for each of the three gene silencing mutants (drm2, drm3, and nrpe1/Pol V catalytic domain). We first downloaded the raw read files from SRA/ NCBI, trimmed and mapped the reads to the *Arabidopsis* reference genome and produced the mtable file for each dataset. The five mtable files were uploaded to TEA and joined to TEA annotation database to build a data analysis website (http://tea.iis.sinica.edu.tw/tair10_demo_new/).

**Fig. 4:**
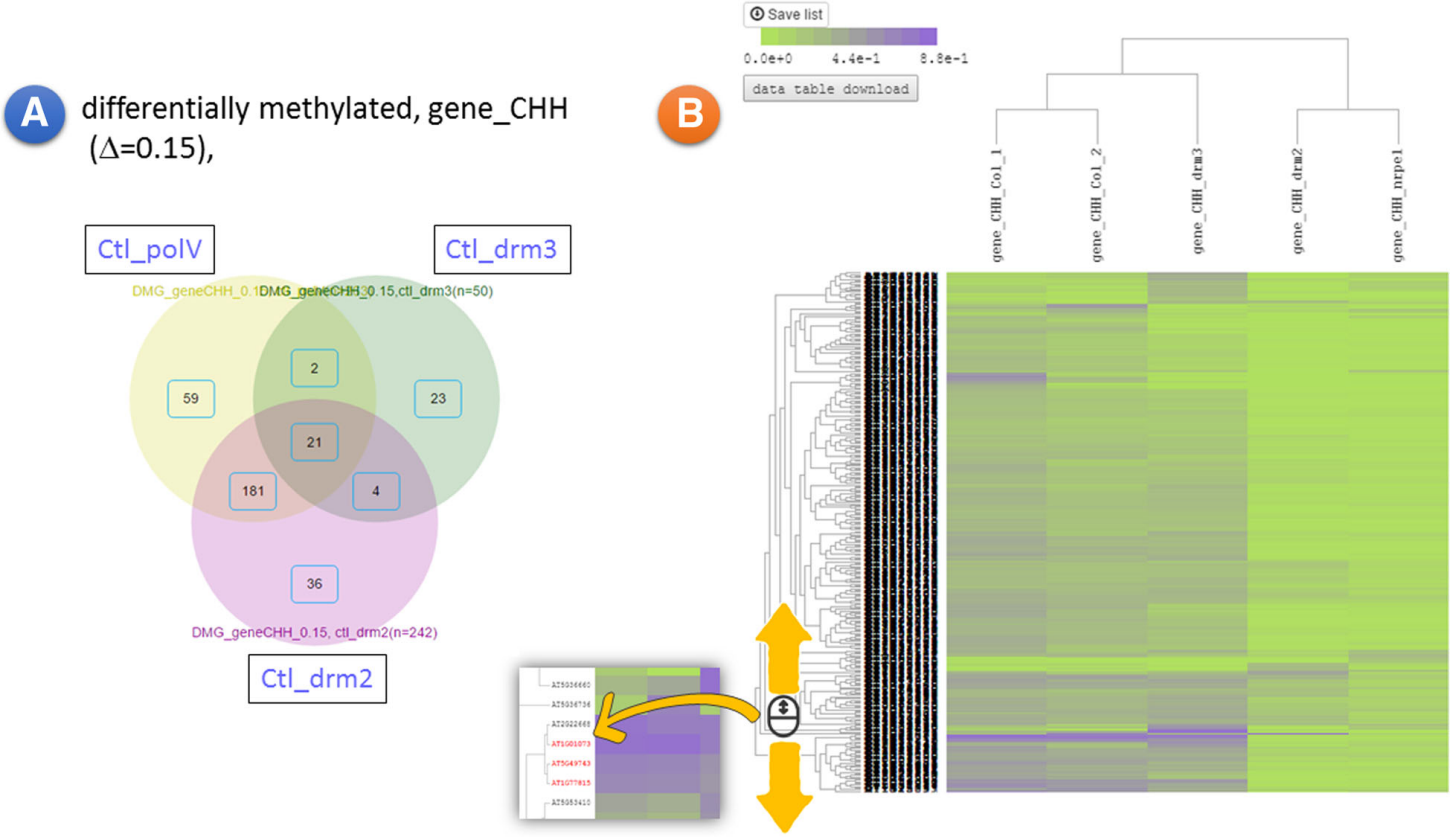
A reanalysis of the case study dataset. **a** Using the criterion of differentially methylated (Δ = 0.15) on the methylation index CHH-gene, DMGs were selected from *drm2, drm3*, and *nrpe1 (polV)* mutants in comparison with the control group. **b** The left panel is a Venn diagram to show the overlap of genes among the three DMG sets. A resizable 2D heat map plot of the union set indicates the profile pattern (similarity) among the five datasets

Firstly, we examined the summary of the five datasets. The percentage on gene id mapping and the usable measurements were acceptable. The methylation measurement data distribution showed a higher level of CG methylation in both gene body and promoter regions, agreeing with the general idea that CG is the major methylation type in the *A. thaliana* genome. Moreover, mutant *drm2* had the most obvious effect (decreased methylation level) on the overall CHH methylation status in both gene body and promoter regions, whereas the *drm3* mutant had the mildest effect. We further examined the gene body CHH methylation level using a criterion of gene body CHH methylation level difference ≥0.15 from the control group (Col-1 + Col-2). Gene lists from the three comparisons performed in the “DMGs” module were analyzed in the module “GeneList Analysis” using the Venn diagram and heatmap plot approaches with 2D clustering (Fig. 4). The control-to-drm3 (Ctl_drm3) DMGs number were the smallest set. The heatmap-2D clustering result indicated that the profile of DMGs selected by gene body CHH level were alike in *drm2* and nrpe1 (polV).

## Discussion

The report of a BS-Seq mapping tool is often a table to count mappings on each C base in the reference genome, numerating the “C” and “C + T” events with or without the sequence context notation. Although the mapping info has been degenerated a lot from the original alignment result, the file size of the mapping report is still hard to handle in a web-based analysis tool. The measurement introduced in this study is a further reduction on the methylation landscape from the single-base level to the gene level. Each measurement is an average of five observations (a particular C context) and each observation is based on at least four events (mapped reads). A deviation of 0.1 in the measurement reflects a change in the methylation state of 10% of the Cs in the observation. Although we cannot tell whether the changes are concentrated in a few sites or more dispersed among the observed sites, using this straightforward approach, we reanalyzed a published dataset and observed the same trends in the methylation landscapes caused by gene silencing.

DMRs identified from different studies are not easily inferred as equivalent because the methods applied are not guaranteed to be compatible to each other. Therefore, a reanalysis of published datasets is necessary for reusing data. It is cost-effective to perform data mining before conducting a new experiment. In a more practical scenario, researchers can try to find a compatible dataset from some metadata depository, such as SRA/NCBI, TAIR, or MPSS [44] and conduct the analysis together with their experimental data to increase the power of the data. An interesting aspect of reanalyzing openly accessed data is to dig out some novel findings not mentioned in the article, because either the authors were not interested in them, or the findings were not covered in the article’s scope.

In summary, we present TEA, a streamlined WGBS analysis platform with versatile analysis and display modules. It provides a straightforward methodology to explore the methylation status in different sequence contexts. The openly accessible TEA provides a dynamic URL for one-month access. Further extension of this work will include more model organisms in the platform and more sophisticated and robust models to select DMGs. To alleviate the burden of upstream BS-seq data alignment, we are going to integrate the preprocessing step into the Galaxy platform.

## Conclusions

TEA is a user-friendly platform for WGBS analysis. It is fast and efficient to select the DMGs because it shrinks the input methylation data from the single-base level to a gene-level methylation profile. It provides several ways to help users exploit and discover their uploaded high-throughput data. It can also facilitate data sharing among cooperators. An unregistered usage is available for creating a working platform with a dynamic URL for one-month access. TEA is freely available for academic users. We welcome researchers to ask for cooperation, keeping the data on a password-controlled access website or an open-access website.

## References

[1] Loenen WA, Dryden DT, Raleigh EA, Wilson GG, Murray NE (2014). Highlights of the DNA cutters: a short history of the restriction enzymes. Nucleic Acids Res.

[2] Furner IJ, Matzke M (2011). Methylation and demethylation of the Arabidopsis genome. Curr Opin Plant Biol.

[3] Suzuki MM, Bird A (2008). DNA methylation landscapes: provocative insights from epigenomics. Nat Rev Genet.

[4] Zemach A, McDaniel IE, Silva P, Zilberman D (2010). Genome-wide evolutionary analysis of eukaryotic DNA methylation. Science.

[5] Bird A (2002). DNA methylation patterns and epigenetic memory. Genes Dev.

[6] Jones PA (2012). Functions of DNA methylation: islands, start sites, gene bodies and beyond. Nat Rev Genet.

[7] Law JA, Jacobsen SE (2010). Establishing, maintaining and modifying DNA methylation patterns in plants and animals. Nat Rev Genet.

[8] Finnegan EJ, Dennis ES (1993). Isolation and identification by sequence homology of a putative cytosine methyltransferase from Arabidopsis thaliana. Nucleic Acids Res.

[9] Finnegan EJ, Peacock WJ, Dennis ES (1996). Reduced DNA methylation in Arabidopsis thaliana results in abnormal plant development. Proc Natl Acad Sci U S A.

[10] Jones L, Ratcliff F, Baulcombe DC (2001). RNA-directed transcriptional gene silencing in plants can be inherited independently of the RNA trigger and requires Met1 for maintenance. Curr Biol.

[11] Ronemus MJ, Galbiati M, Ticknor C, Chen J, Dellaporta SL (1996). Demethylation-induced developmental pleiotropy in Arabidopsis. Science.

[12] Vongs A, Kakutani T, Martienssen RA, Richards EJ (1993). Arabidopsis thaliana DNA methylation mutants. Science.

[13] Du J, Zhong X, Bernatavichute YV, Stroud H, Feng S, Caro E, Vashisht AA, Terragni J, Chin HG, Tu A (2012). Dual binding of chromomethylase domains to H3K9me2-containing nucleosomes directs DNA methylation in plants. Cell.

[14] Jackson JP, Lindroth AM, Cao X, Jacobsen SE (2002). Control of CpNpG DNA methylation by the KRYPTONITE histone H3 methyltransferase. Nature.

[15] Lindroth AM, Cao X, Jackson JP, Zilberman D, McCallum CM, Henikoff S, Jacobsen SE (2001). Requirement of CHROMOMETHYLASE3 for maintenance of CpXpG methylation. Science.

[16] Stroud H, Do T, Du J, Zhong X, Feng S, Johnson L, Patel DJ, Jacobsen SE (2014). Non-CG methylation patterns shape the epigenetic landscape in Arabidopsis. Nat Struct Mol Biol.

[17] Matzke MA, Kanno T, Matzke AJ (2015). RNA-directed DNA methylation: the evolution of a complex epigenetic pathway in flowering plants. Annu Rev Plant Biol.

[18] Matzke MA, Mosher RA (2014). RNA-directed DNA methylation: an epigenetic pathway of increasing complexity. Nat Rev Genet.

[19] Hebestreit K, Dugas M, Klein HU (2013). Detection of significantly differentially methylated regions in targeted bisulfite sequencing data. Bioinformatics.

[20] Hansen KD, Langmead B, Irizarry RA (2012). BSmooth: from whole genome bisulfite sequencing reads to differentially methylated regions. Genome Biol.

[21] Stockwell PA, Chatterjee A, Rodger EJ, Morison IM (2014). DMAP: differential methylation analysis package for RRBS and WGBS data. Bioinformatics.

[22] Song Q, Decato B, Hong EE, Zhou M, Fang F, Qu J, Garvin T, Kessler M, Zhou J, Smith AD (2013). A reference methylome database and analysis pipeline to facilitate integrative and comparative epigenomics. PLoS One.

[23] Akalin A, Kormaksson M, Li S, Garrett-Bakelman FE, Figueroa ME, Melnick A, Mason CE (2012). methylKit: a comprehensive R package for the analysis of genome-wide DNA methylation profiles. Genome Biol.

[24] Kishore K, de Pretis S, Lister R, Morelli MJ, Bianchi V, Amati B, Ecker JR, Pelizzola M (2015). methylPipe and compEpiTools: a suite of R packages for the integrative analysis of epigenomics data. BMC Bioinformatics.

[25] Park Y, Figueroa ME, Rozek LS, Sartor MA (2014). MethylSig: a whole genome DNA methylation analysis pipeline. Bioinformatics.

[26] Sun D, Xi Y, Rodriguez B, Park HJ, Tong P, Meong M, Goodell MA, Li W (2014). MOABS: model based analysis of bisulfite sequencing data. Genome Biol.

[27] Dolzhenko E, Smith AD (2014). Using beta-binomial regression for high-precision differential methylation analysis in multifactor whole-genome bisulfite sequencing experiments. BMC Bioinformatics.

[28] Liang F, Tang B, Wang Y, Wang J, Yu C, Chen X, Zhu J, Yan J, Zhao W, Li R (2014). WBSA: web service for bisulfite sequencing data analysis. PLoS One.

[29] Lamesch P, Berardini TZ, Li D, Swarbreck D, Wilks C, Sasidharan R, Muller R, Dreher K, Alexander DL, Garcia-Hernandez M (2012). The Arabidopsis Information Resource (TAIR): improved gene annotation and new tools. Nucleic Acids Res.

[30] Kim MY, Zilberman D (2014). DNA methylation as a system of plant genomic immunity. Trends Plant Sci.

[31] Stroud H, Greenberg MV, Feng S, Bernatavichute YV, Jacobsen SE (2013). Comprehensive analysis of silencing mutants reveals complex regulation of the Arabidopsis methylome. Cell.

[32] Guo W, Fiziev P, Yan W, Cokus S, Sun X, Zhang MQ, Chen PY, Pellegrini M (2013). BS-Seeker2: a versatile aligning pipeline for bisulfite sequencing data. BMC Genomics.

[33] Krueger F, Andrews SR (2011). Bismark: a flexible aligner and methylation caller for Bisulfite-Seq applications. Bioinformatics.

[34] Yong WS, Hsu FM, Chen PY (2016). Profiling genome-wide DNA methylation. Epigenetics Chromatin.

[35] Bernstein BE, Stamatoyannopoulos JA, Costello JF, Ren B, Milosavljevic A, Meissner A, Kellis M, Marra MA, Beaudet AL, Ecker JR (2010). The NIH roadmap epigenomics mapping consortium. Nat Biotechnol.

[36] Consortium EP (2012). An integrated encyclopedia of DNA elements in the human genome. Nature.

[37] Adams D, Altucci L, Antonarakis SE, Ballesteros J, Beck S, Bird A, Bock C, Boehm B, Campo E, Caricasole A (2012). BLUEPRINT to decode the epigenetic signature written in blood. Nat Biotechnol.

[38] [http://ihec-epigenomes.org/] Accessed on May, 2016.

[39] Down TA, Piipari M, Hubbard TJ (2011). Dalliance: interactive genome viewing on the web. Bioinformatics.

[40] Krzywinski M, Schein J, Birol I, Connors J, Gascoyne R, Horsman D, Jones SJ, Marra MA (2009). Circos: an information aesthetic for comparative genomics. Genome Res.

[41] Zhong X, Hale CJ, Nguyen M, Ausin I, Groth M, Hetzel J, Vashisht AA, Henderson IR, Wohlschlegel JA, Jacobsen SE (2015). Domains rearranged methyltransferase3 controls DNA methylation and regulates RNA polymerase V transcript abundance in Arabidopsis. Proc Natl Acad Sci U S A.

[42] Greenberg MV, Deleris A, Hale CJ, Liu A, Feng S, Jacobsen SE (2013). Interplay between active chromatin marks and RNA-directed DNA methylation in Arabidopsis thaliana. PLoS Genet.

[43] Martin M. Cutadapt removes adapter sequences from high-throughput sequencing reads. EMBnet J. 2011;17(1):10-12.

[44] Li P, Demirci F, Mahalingam G, Demirci C, Nakano M, Meyers BC (2013). An integrated workflow for DNA methylation analysis. J Genet Genomics.

